# Idunium

**Published:** 1885-02

**Authors:** 


### Idunium.

"Idunium" is the name proposed by Professor Websky for the metal just discovered by him as one of the components of native vanadate of lead. The mineral is rather a scarce one of a yellow color, and contains several other metals, of which zinc, iron, and arsenic are among the most prominent. Idunium resembles vanadine in several respects, both physically and chemically, while the only oxide hitherto examined forms stable salts with alkaline bases, and thus would appear to possess distinctly acid properties. It will probably be known by and by as "idunic acid," and as its general characteristics and reactions correspond to those of vanadic acid, its formula will probably be $Id_2O_5$.

In England, recently, whilst a dentist was complying with a patient's request to use his painless dentistry process, in which bichloride of methylene was used, he observed that the boy's head dropped, that he turned pale, and that he was gasping for breath. A restorative was administered, but without success, and a medical man was summoned, but before his arrival death had occurred.

